# Conditioned Medium of Wharton's Jelly Derived Stem Cells Can Enhance the Cartilage Specific Genes Expression by Chondrocytes in Monolayer and Mass Culture Systems

**DOI:** 10.15171/apb.2017.016

**Published:** 2017-04-13

**Authors:** Maryam Hassan Famian, Soheila Montazer Saheb, Azadeh Montaseri

**Affiliations:** ^1^Department of molecular biology, Ahar Branch, Islamic Azad University, Ahar, Iran.; ^2^Stem Cell Research Center, Tabriz University of Medical Sciences, Tabriz, Iran.

**Keywords:** Osteoarthritis, Mesenchymal stem cells, Wharton's jelly, Conditioned medium

## Abstract

***Purpose:*** Mesenchymal stem cells (MSCs) have been introduced for cell therapy strategies in osteoarthritis (OA). Despite of their capacity for differentiation into chondrocyte, there are some evidences about their life-threatening problem after transplantation. So, some researchers shifted on the application of stem cells conditioned medium. The goal of this study is to evaluate whether Wharton's jelly derived stem cell conditioned medium (WJSCs-CM) can enhance the gene expression profile by chondrocytes in monolayer and mass culture systems.

***Methods:*** Conditioned medium was obtained from WJSCs at fourth passage. Isolated chondrocytes were plated at density of 1×10^6^ for both monolayer and high density culture. Then cells in both groups were divided into control (received medium) and experiment group treated with WJ-CM for 3 and 6 days. Samples were prepared to evaluate gene expression profile of collagen II, aggrecan, cartilage oligomeric matrix protein (COMP) and sox-9 using real-time RT-PCR.

***Results:*** After 3 days, Chondrocytes treated with WJSCs-CM expressed significantly higher level of genes compared to the control group in both culture systems. After 6 days, the expression of genes in monolayer cultivated chondrocytes was decreased but that of the mass culture were up-regulated significantly.

***Conclusion:*** WJ-SCs-CM can increase the expression of cartilage-specific genes and can be introduced as a promoting factor for cartilage regeneration.

## Introduction

Articular cartilage which covers the joint surfaces is a highly specialized type of connective tissue that predominantly composed of one cell type named chondrocytes scattered in a lattice of extracellular matrix (ECM). In normal cartilage, chondrocytes are responsible for control of constant degradation and re-synthesis of ECM components such as collagen II, aggrecan and cartilage oligomeric matrix protein (COMP).^1^ Due to the lack of blood vessels, articular cartilage has limited capacity for self-repair and if its lesions left untreated, consequently progresses toOsteoarthritis (OA).^2^ The orthopedic diseases such as Rheumatoid Arthritis (RA) and OA are the major causes of disability and affect about 250 million people worldwide.Osteoarthritis which is the most common form of rheumatoid arthritis, is an active pathological process characterized by ECM degradation that consequently results in joint stiffness, mobility restriction, subchondral bone sclerosis and finally disability.^3,4^ The etiology of OA is unknown, but it can occur due to different factors such as aging, obesity, joint trauma and genetic susceptibility.^5^ The metabolism of normal articular cartilage is maintained as a result of a delicate balance between synthesis and degradation of ECM components which is regulated by chondrocytes.^1^ At the molecular level, onset of OA occurs due to impairment of this precisely controlled mechanism.^6^ It has been proved that pro-inflammatory cytokines such as Interleukin-1β (IL-1β) and Tumor necrosis factor-α (TNF-α) which are secreted by synoviocytes and chondrocytes enhance the synthesis of matrix degrading enzymes such as matrix metaloproteinases (MMPs) resulting in collagen and proteoglycan loss‏.^7^ On the other hand, in osteoarthritic cartilage, absence of anabolic growth factors such as TGF-β, BMPs and IGF-1 can make this tissue more susceptible to damage.^1,5^ A great number of investigations explored the pivotal roles of these growth factors in acceleration of cartilage formation and integration.^1,8^ TGF-β family members have vital function in cell proliferation, migration, control of ECM synthesis, degradation and also have a very important role in cartilage development.^9,10^ Different investigations revealed that enhancement of cartilage repair in *in vivo* conditions, increased proteoglycan synthesis, stimulation of mesenchymal stem cell differentiation into chondrocytes and maintenance of differentiated state of these cells can be occurred in the presence of TGF-β superfamily members.^11,12^ Another growth factor with anabolic effects in articular cartilage is insulin like growth factor-1 (IGF-1).^13^ It promotes repair of cartilage defects, is able to strongly stimulate matrix synthesis and reverses the catabolic effects of pro-inflammatory cytokines through suppression of IKB-α kinase.^8,14^ Recently, medical research focus shifted to the application of stem cells in order to reduce a number of debilitating diseases such as musculoskeletal disorders.^15^ During last few years, interest in cell implantation strategies to restore the impaired cartilage has emerged. Autologous chondrocyte implantation (ACI) is the most common cell-based surgical method, but it has some disadvantages such as further injury to the healthy cartilage and *in vitro* dedifferentiation of chondrocytes which is occurred due to increasing the passage numbers required for obtaining sufficient cells for implantation.^16^ Compared to the chondrocytes, Mesenchymal stem cells (MSCs) can be isolated and expanded easier with less donor morbidity, are available in large quantities and have the capacity to differentiate into chondrocytes.^17,18^ Human umbilical cord (UC) is a rich source for MSCs with characteristics typical to the bone-marrow derived stem cells.^19^ This postnatal tissue is easily accessible because normally discarded after birth, so it’s a noncontroversial source for MSCs. The mucoid Wharton’s jelly also known as intervascular UC tissue, composed of fibroblast-like cells recognized as multipotent MSCs capable to differentiate into chondrocytes in* in vitro* and *in vivo* conditions.^20,21^ It has been reported that Wharton’s jelly mesenchymal stem cells (WJ-MSCs) can up-regulated the synthesis of cartilage ECM molecules such as hyalorunic acid and glycosaminoglycans and also enhance the SOX-9, COMP, and type II collagen gene expression.^22^ Despite the regenerative capacity of Mesenchymal stem cells, normally engrafted cells have poor differentiation and survival rates and it has been reported that administration of this cells for clinical application can induce the risk of cancer. So, it’s necessary to find a solution for this hazardous and life-threatening problem.^23^ Stem cells secrete a wide spectrum of elements such as trophic, anti-apoptotic and immunomodulatory factors into culture medium (CM) through paracrine activity,^4,23,24^ so it seems that stem cell-CM may overcome the obstacles of using them alone.

Taken together, regarding to the presence of trophic factors in the CM of WJ-SCs and the role of them in promotion of cartilage metabolism, the goal of the present study is to investigate whether WJ-SCs-CM can promote the expression of cartilage specific genes such as collagen type II, SOX-9, COMP andaggrecan by chondrocytes.

## Materials and Methods

### Chondrocyte isolation and culture

Cartilage samples were taken from patients undergoing joint replacement surgery for femoral neck fractures. All patients gave written informed consent andthe institutional review board and medical ethics committee of Tabriz University of Medical Sciences approved the study protocol. For chondrocyte isolation, after 3 times washing with PBS, cartilage samples were cut into 1×1 mm thick pieces using sterile scalpel and then incubated in 1% pronase enzyme for 1 hour. In the next step, samples were incubated by collagenase type II (0.02% solved in medium) in a shaking water bath at 37°^C^ for 4-6 hours. The digested samples were centrifuged at 1500 rpm/5 min and obtained chondrocytes were seeded at 5×10^5^ cells per T75 flasks and incubated at 37°^C^/ co_2_ 5%. The first medium change was performed after 24 hours and following medium changes were performed three times per week. After reaching about 70% confluency, cells were passaged.

### Harvesting the mesenchymal stem cells from Wharton^’^s jelly

Umbilical cord samples were obtained from women who underwent caesarean section (S/C)‏. Written consent was obtained from all patients and institutional ethical review board of Tabriz University of Medical Sciences, Tabriz, Iran approved the study protocol. Samples were stored aseptically in cold PBS containing antibiotics and transported to the cell culture lab. After 3 times washing, umbilical cord samples inserted into 70% ethanol for 30 seconds and then cut into 2.5-3 cm long pieces and placed in a sterile dish. Then, using a scissor, cord pieces were incised longitudinally and vessels were dissociated. In the next step, delicate Wharton^’^s jelly (WJ) tissue was separated from amniotic layer and later cut into small fragments (1×2 mm) using scalpel. Obtained WJ samples were placed in the T-25 culture flasks with Dulbeco's Modified Eagle Medium (DMEM)containing 20% fetal bovine serum (FBS) and penicillin/streptomycin (P/S) 1%. After about 14 days, stem cells started to crawl from explanted WJ tissues. It should be noted that such cells have been characterized as mesenchymal stem cells by identification of specific cell surface markers on these cells in studies performed by our coworkers.^25^ When migrated cells filled about 70% of culture flask, subculture was performed using Trypsin/EDTA (0.05%).

### Preparation of supernatant from Wharton^’^s jelly derived stem cell

Fourth passage Wharton^’^s jelly derived mesenchymal stem cells (WJSCs) were used for conditioned medium (supernatant) collection. After reaching about 70% confluency, the supernatant was discarded and the attached cells were washed with PBS‏. Then, serum-free DMEM was added and cells were incubated for 48 hours. Obtained conditioned medium was centrifuged at 1500 rpm for 5 min for removing any debries. The second centrifugation was done at 3000 rpm for 3 min and samples were stored at -80°^C^till further use. Furthermore to compare the secretion of growth factors by WJ-SCs in different passages, cells at passage 1, 2 and 3 were also obtained as described.

### Experimental design

Chondrocytes at third passage were used for this evaluation. To understand the effect of WJSC-CM on chondrocytes, cells were seeded at density of 1×10^6^ and further divided into control group which received only DMEM culture medium (containing 0.05% FBS) or treated with WJSC-CM for 3 and 6 days. We also evaluated the probable effects of WJ-SCs-CM on chondrocytes cultured in mass culture condition. For this purpose, 1×10^6^ millions of cells were centrifuged in 15 ml conical tubes to form cell pellet. Mass-cultivated chondrocytes were also divided into control and treated groups as described for monolayer cells. After this period, samples were prepared for evaluation of the expression of specific cartilage genes, including collagen type II, aggrecan, COMP and sox9 using Real-time PCR.

### Enzyme – linked immunosorbent assay (ELISA) for measurement of TGF-β1 and IGF-1 in WJ-SCs-CM:

The concentration of TGF-β1 and IGF-1 in the supernatant of WJSCswas measured by ELISAusing Human TGF-β1 ELISA kit (BOSRER, cat. No: EK0513,CA) and Human IGF-BP-1 ELISA kit (BOSRER, cat. No: EK0382,CA).

According to the manufactur^'^s instruction, samples and standards were incubated at 37°^C^ for 90 min. after addition of biotinylated antibodies (60 min), samples were washed using 0.01 M PBS. In the next step, samples were incubated with Avidin-Biotin-Peroxidase complex, following by addition of TMB color developing agent in dark place for 25-30 min. Finally, absorbance was measured at 450 nm using a microplate reader (ELISA Reader, Tecan, CH-8708, Australia)‏.

### Real time RT-PCR

The genetic information for type IIcollagen, Sox-9, aggrecan and cartilage oligomeric matrix protein (COMP) in samples of both control and CM-treated groups was detected by Real-time reverse transcriptase polymerase chain reaction (Real time RT-PCR). Total cellular RNA was extracted using YTA mini kit (Cat.NO: YT9065, Taiwan) according to the manufacture’s protocol. Briefly, after centrifugation and supernatant removal, RB Buffer was added to the cell pellet to lyse the cells. Then sample mixture was transferred to the collection tube containing filter column and centrifuged at 14000 rpm/2 min. In the next step, samples were mixed with 70% ethanol. RB mini column was washed at first with wash buffer 1 and subsequently with wash buffer 2. Finally, RNase–free ddH_2_O was added to the samples and centrifugation at 14000 rpm/2 min was performed to elute RNA. Approximately 1000ng/1 ml of total RNA was used as template for cDNA synthesis using reverse transcription kit (Takara, RR037I, Japan). The Real time RT-PCR reactions were performed using a (Corbett, 010755, Australia) system with a SYBR Green master mix (Takara, RR820L, Japan) under the condition of 15s at 95°^C^ and different time and annealing temperature for each gene as can be found in Table 1. The primer sequences used for this investigation are also listed in Table 1. The gene expression levels were calculated using Pfaffl formula and β-actin used as internal control. All experiments were done in triplicate.

**Table 1:** Primer sequences used in Real Time RT-PCR and related annealing temperature.

**gene**	**primer**	**Annealing temperature**
Sox9-F	AGAGAGGACCAACCAGAATTC	57°c for 30 sec
Sox9-R	TGGGTAATGCGCTTGGATAG	57°c for 30 sec
Coll2-F	GGCAATAGCAGGTTCACGTACA	‏°59c for 30 sec
Coll2-R	CGATAACAGTCTTGCCCCACTT	‏°59c for 30 sec
Comp-F	TGCAATGACACCATCCCAG	56°c for 30 sec
Comp-R	ACACACACTTTATTTTGTCCTCTC	56°c for 30 sec
ACAN-F	CAACTACCCGGCCATCC	56°c for 30 sec
ACAN- R	GATGGCTCTGTAATGGAACAC	56°c for 30 sec
B actin-F	TCCTCCCTG GAG AAG AGC TA	58°c for 45 sec
B actin-R	TCA GGAGGA GCA ATG ATC TTG	58°c for 45 sec

### Statistical analysis

All data are reported as means ± SD. Statistical difference between two groups was determined by Two-way ANOVA and followed by t-test post test. P<0.05 was set as significant.

## Results

### Enhancement of TGF-β and IGF-1 secretion by increased number of WJ-SCs passages

**Figure 1:**
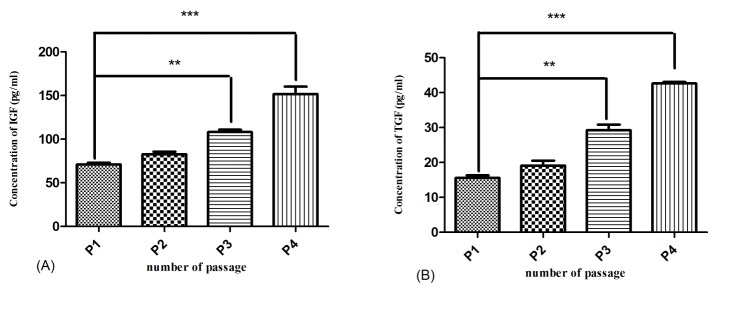
Measurement of IGF-1 (A) and TGF-β (B) secretion by WJ-SCs in different cell passages. **P<0.001, ***P<0.0001

WJSCs at 4 different cell passages cultured in DMEM medium lacking supplemental serum for 48 hours, then after the amount of TGF-β and IGF-1 were measured. As it can be found from Figure 1 (A and B), by increasing the cell passage number, the level of these growth factors secreted by WJSCs enhanced. There is a significant increase in TGF-β and also IGF-1 concentration between WJSCs at third and fourth passage when compared to the first passage of cells, with P-value of less than 0.001 and 0.0001, respectively.

### Effect of WJSCs on chondrocyte gene expression

**Figure 2:**
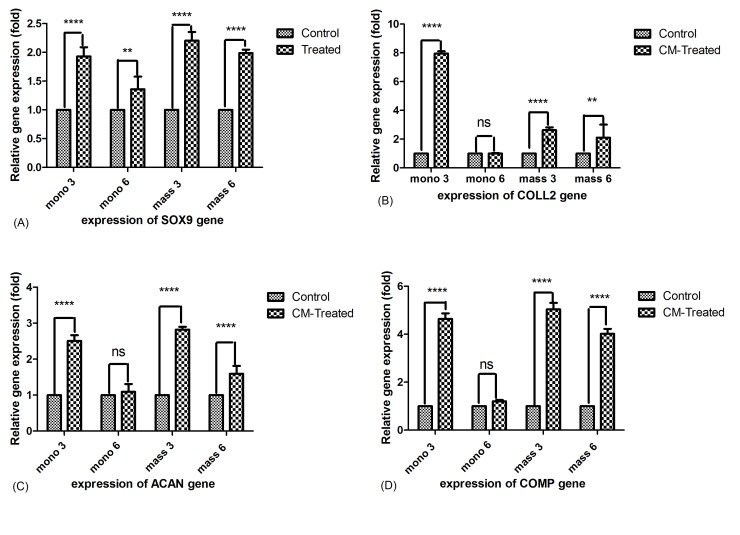
Comparison of cartilage specific gene expression in monolayer and mass cultured chondrocytes on 3 and 6 days. A) Sox-9, B) Collagen type II, C) Aggrecan and D) COMP. Ns: non-significant, **P<0.001, ****P<0.00001

The mRNA expression level of cartilage-specific genes including collagen type II, Sox-9, Aggrecan and COMP was assessed in monolayer and mass-cultured chondrocytes treated with WJ-SCs-CM for 3 and 6 days. As it has been revealed in Figure 2 (A-D) the expression of all of the above mentioned genes was up-regulated significantly in monolayer chondrocytes after 3 days compared to the control (P<0.00001).There is no significant difference between treated and control chondrocytes in monolayer after 6 days of culture period in the expression of collagen II, aggrecan (ACAN) and COMP genes but that of SOX-9 up-regulated on 6 days of culture period, too (P<0.001) as can be observed in Figure 2 (A).

In mass cultivated chondrocytes, the expression of Sox-9, Collagen II, aggrecan and COMP genes was enhanced significantly in chondrocytes treated with WJ-SCs-CM compared to the control cells on both time points as can be found from Figure 2 (A-D).

**Figure 3:**
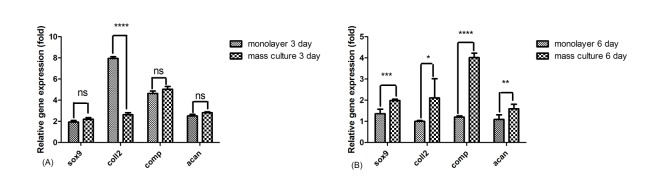
The analysis of the expression of cartilage-specific genes in the chondrocytes cultured in monolayer and mass culture on A) 3 days and B) 6 days of culture period. Ns: non-significant, *p<0.01, **p<0.001, ***p<0.0001 and ****p<0.00001

On the third day of the experiment, as it can be understood from Figure 3 (A) there is no significant difference in expression of Sox-9, aggrecan and COMP in monolayer and mass cultured chondrocytes treated with WJ-SCs-CM, but that of the Collagen type II was significantly increased in monolayer cells in comparison to the mass culture (P<0.00001). After 6 days of culture periodas described in Figure 3 (B)the expression level of these genes increased significantly in mass cultured chondrocytes treated with WJ-SCs-CM compared to the treated chondrocytes in monolayer culture.

## Discussion

In this study we found that exposure of chondrocytes to the WJ-SCs conditioned medium resulted in up-regulation of cartilage specific genes such as aggrecan, COMP, SOX-9 and Collagen type II in both monolayer and mass culture systems on the third day of culture period, but after 6 days, the expression of these genes up-regulated only in mass culture chondrocytes.

Osteoarthritis is the major cause of disability in the elderly that occurs mostly due to an imbalancebetween synthesis and degradation of cartilage ECM‏.^26^ Strategies for application of mesenchymal stem cells (MSCs) as a feasible cell-based therapy are being investigated for regenerative medicine‏.^27^ MSCs can be obtained from various sources and because of their chondrogenic potential, considered as a hopeful candidate in cartilage regenerative medicine‏.^28^ Stem cells derived from umbilical cord Wharton’s jelly show high proliferative capacity, can differentiate into the three germ lineages, are available in large numbers and have prolonged stemness characteristic‏.^29,30^ These advantages combined with their non-controversial nature and non-tumorogenicity make them attractive cell source for cell-based therapy‏.^15^ The exact mechanism by which WJ-SCs exert their function isn’t yet completely known but they can affect the other cell’s behavior through secretion of specific molecules such as cytokines and growth factors‏.^27,30^ For the clinical application of stem cells, it is necessary to produce these cells under good manufacturing practice and control their biosafety and purity‏.^27^ Recently cell-free based therapy has been introduced to overcome these limitations.

In this study we investigated whether WJ-SCs-CM can enhance the gene expression profile by chondrocytes. The results of this study demonstrate that WJ-SCs-CM can promote the expression of aggrecan, COMP, Collagen type II and SOX-9 in both time points after 3 days.

The master transcription SRY-BOX 9 (SOX-9) which is expressed in chondroprogenitors and mature chondrocytes, is an essential factor for chondrocyte differentiation‏.^31,32^ SOX-9 regulates the synthesis of cartilage ECM components such as collagen type II and aggrecan and also suppresses the chondrocyte hypertrophy‏.^32,33^ In this study we showed that in the presence of WJ-SCs-CM the expression of sox-9 gene can be up-regulated. The increased expression of sox-9 can explain the up-regulation of other ECM components genes by chondrocytes treated with WJ-SCs-CM.

In this study we also found that treatment of chondrocytes with conditioned medium of WJ-SCs increased the expression of collagen type II gene as an essential element of articular cartilage, on the third day of culture in monolayer chondrocyte but not at the 6 days of culture period. This type of collagen gives tensile strength and provides most of the mechanical properties of cartilage tissue‏.^21,34^ Decreased level of collagen type II by chondrocytes in monolayer condition after 6 days can be contributed to the loss of phenotype of this cells by increasing the time. Cartilage Oligomeric Matrix Protein (COMP) is a non-collagenous protein found in territorial matrix surrounding the chondrocytes and regulates the collagen network in cartilage tissue‏.^35^ Another component of articular cartilage ECM is aggrecan that donates resilience and flexibility to this tissue. During OA process the ECM molecules are degraded enzymatically, so the restoration and repair of articular cartilage requires the re-construction of ECM by synthesis of these macromolecules‏.^36^

As it has been previously described, the metabolism of normal articular cartilage is regulated by different anabolic growth factors^8^ and decrease in these stimuli will disrupt the cartilage integrity‏.^1^ An example for these anabolic stimuli is TGF- β which is expressed in high levels in normal cartilage tissue and have an important role in maintaining the chondrocyte phenotype, ECM synthesis and enhancing the biochemical composition of articular cartilage‏.^5,37^ IGF-1 is another major factor involved in modulating the collagen network, increasing the proteoglycan and collagen synthesis and inhibiting the matrix degradation rate of cartilage tissue‏.^38,39^ In this study we found that the anabolic growth factors including TGF-β and IGF-1 were secreted into the supernatant of WJ-SCs after 48hr of serum starvation and the increased expression of cartilage-specific genes by chondrocytes can be contributed to these factors. Serum deprivation stimulates MSCs for secretion of different factors that are necessary for cell survival and antagonization of toxic condition so it can promote the effectiveness of MSC-CM through higher amount of trophic factors secreted by these cells‏.^24^ As a consequent, application of conditioned medium can bring the paracrine secretion of stem cells without considerations of immune system responses or tumor formation of stem cell transplantation.^23,40^

It has been widely accepted that chondrocyte phenotype is lost during long *in vitro* monolayer culture so culturing of chondrocytes in 3D condition is suggested to support the re-differentiation of these cells‏.^41^ In this study we also used the mass culture of chondrocytes in which the cell-cell and cell-matrix interactions can be established better and then we treated them with WJ-SCs-CM as in monolayer group. It has been revealed for us that the cultivated chondrocytes in mass culture can express SOX-9, Collagen II, aggrecan and COMP in significantly higher amount compared to the monolayer condition. So, the high-density (mass) culture system employed in this study seems to be a prosperous method for retention of chondrocyte phenotype compared to the monolayer culture.

During the process of chondrogenesis, condensation of chondrogenic cells provides a strong cell-cell contact that can be mimicked using mass culture technique‏.^42^ Furthermore the expression of SOX-9 by highly packed chondroprogenitor cells enhance during this phase of chondrogenesis that eventually results in the regulation of cartilage specific matrix components such as collagen II and aggrecan‏.^43^ In line with these data, our study also reported that the expression of chondrocyte genes was significantly higher in mass cultivated cells compared to the monolayer condition when treated with WJ-SCs-CM.

## Conclusion

The results presented in this investigation suggest that WS-SCs conditioned medium is a potent, safe and relatively cost-benefit medium for enhancing the expression of cartilage specific genes in both monolayer and mass culture system. Findings of this study also revealed that the expression of genes by WJ-SCs-CM treated chondrocytes in mass culture condition is stronger when compared to the treated monolayer cells.

## Acknowledgments

This article is resulted from the research proposal leading to thesis of Maryam Hassan Famian, M.Sc student of genetics and approved by Stem Cell Research center, Tabriz University of Medical sciences, Tabriz, Iran. The authors gratefully acknowledge the research deputy of Tabriz Universityof Medical Sciences for financial support.

## Ethical Issues

Not applicable.

## Conflict of Interest

The authors declare no conflict of interests.
